# IUC22936-87 Evaluating negative biopsy outcomes for suspicious bladder red patches—are we over-performing biopsy? A 2-cycle audit

**DOI:** 10.1093/oncolo/oyaf248.006

**Published:** 2025-08-29

**Authors:** A Hossain, P Papikinos, K Botond, A Chandrakumaran

**Affiliations:** Surrey and Sussex Healthcare, NHS Trust, United Kingdom; Surrey and Sussex Healthcare, NHS Trust, United Kingdom; Surrey and Sussex Healthcare, NHS Trust, United Kingdom; Surrey and Sussex Healthcare, NHS Trust, United Kingdom

## Abstract

**Background:**

Red patches identified during flexible cystoscopy are often biopsied due to concerns of carcinoma in situ (CIS) or urothelial malignancy. However, there is limited guidance on when to biopsy these lesions, leading to potential over-treatment. To evaluate the clinical value of performing bladder biopsies for red patches identified during flexible cystoscopy and to determine the proportion of negative histological outcomes. This audit aimed to assess whether current practice results in overperformance of biopsies, particularly in patients without high-risk features.

**Methods:**

This retrospective 2-cycle audit was conducted at the District General Hospital in the UK. In Cycle 1 (March-June 2024), and Cycle 2 (November 2024-February 2025), all patients undergoing flexible cystoscopy were screened for suspicious red patches. Relevant cases were identified through endoscopy logs and electronic records. Data collected included patient demographics, presenting symptoms, reason for cystoscopy, decision pathway (relook vs biopsy), and histological outcomes. During Cycle 2, a total of 1,168 flexible cystoscopies were performed—937 by urology registrars and 24 by specialist nurses.

**Results:**

In Cycle 2, 75 patients were identified with red bladder patches during flexible cystoscopy. Of these, 33 patients (44%) proceeded to rigid cystoscopy and biopsy, with only 1 case (3%) confirming malignancy; all others were benign or inflammatory. The remaining 49 patients (65%) underwent relook flexible cystoscopy within 2-4 weeks; in 39 (80%) of these cases, the red patches had resolved, avoiding rigid cystoscopy and biopsy. Following Cycle 1, a new practice was implemented allowing clinicians and trained specialty nurses to capture cystoscopic images of red patches and upload them to the Cerner electronic system for review by consultants or senior registrars. This promoted senior input in biopsy decision-making and helped standardize management. Across both cycles, biopsy request rates remained low—4% in Cycle 1 and 3% in Cycle 2—supporting a selective, risk-stratified approach.

**Conclusions:**

The diagnostic yield of biopsies for red bladder patches following flexible cystoscopy is low, particularly in patients without high-risk features. Our findings support a more selective approach, where relook flexible cystoscopy and clinical reassessment help avoid unnecessary rigid procedures.

